# Economy-wide impact of a reduction in tobacco use in India

**DOI:** 10.1136/tc-2023-058471

**Published:** 2024-09-27

**Authors:** Rijo M John, Badri Narayanan, Sumathi Chakravarthy, Sindhu Bharathi, Praveen Sinha, Vineet Gill Munish, Mark Goodchild

**Affiliations:** 1 Rajagiri College of Social Sciences, Cochin, Kerala, India; 2 Boston College, Chestnut Hill, Massachusetts, USA; 3 Infinite Sum Modelling LLC, Seattle, Washington, USA; 4 World Health Organization, New Delhi, India; 5 World Health Organization, Geneva, Switzerland

**Keywords:** Economics, Low/Middle income country, Taxation, Public policy

## Abstract

**Background:**

Public policy measures aimed at regulating tobacco use should consider the net gains for the nation, as the tobacco sector contributes to employment and tax revenue while also imposing substantial economic burden on the country. This study investigates the economy-wide impact of reducing tobacco consumption in India through the implementation of fiscal measures.

**Methods:**

The study uses a computable general equilibrium model based on the Global Trade Analysis Project model and database and augments the same with several country-specific information on tobacco products, to examine the macroeconomic impact of a targeted reduction in the consumption of bidis, cigarettes and smokeless tobacco by 10% by the year 2026 through the adoption of fiscal measures.

**Results:**

The model results suggest that the targeted reduction in consumption may result in a 0.14% reduction in the gross domestic product (GDP) and a 0.44% reduction in overall employment in the economy. However, after accounting for the averted premature deaths due to tobacco use, the results indicate a net 0.22% increase in GDP and a net increase in employment of about 1.36 million jobs (or 0.29% of the labour force) over 5 years. Further, the tax increase measures proposed in this model to achieve the targeted reduction in consumption would generate an additional US$2774 million in revenues to the exchequer.

**Conclusion:**

The impact of targeted prevalence reduction of tobacco use is a win-win for the country considering its positive macroeconomic impacts in terms of net increases in both GDP as well as employment.

**WHAT IS ALREADY KNOWN ON THIS TOPIC**

- Due to the contributions of the tobacco sector to both employment and tax revenue in India, debates on fiscal policies aimed at regulating this industry are often intensified. Arguments frequently emphasise the economic impact of the tobacco industry, highlighting concerns about potential job losses and reduced tax contributions.

**WHAT THIS STUDY ADDS**

- Using a computable general equilibrium model, this study assesses the broad economic impacts of decreasing tobacco consumption in India through fiscal measures. The findings reveal that, considering the prevention of premature deaths from tobacco use, there is a net positive effect, with a 0.22% increase in gross domestic product and an addition of around 1.36 million jobs over 5 years. Moreover, the suggested tax increase measures in the model could generate an additional US$2774 million in revenue for the exchequer.

**HOW THIS STUDY MIGHT AFFECT RESEARCH, PRACTICE OR POLICY**

- This study underscores the comprehensive economic benefits associated with enforcing tobacco control measures. It suggests that fiscal measures for tobacco regulation can be crafted with a broader macroeconomic outlook, offering insights for policy-makers. The innovative methodology applied in this research presents potential value for replication in other countries heavily reliant on the tobacco sector.

## Introduction

 India, with 267 million people above 15 years of age using tobacco in some form^1^ in 2017, is the second-largest consumer after China and constitutes approximately 19% of worlds’ adult tobacco users.^2^ India was also the third-largest producer of tobacco after China and Brazil in 2018 accounting for 12.3% of the world’s tobacco production.^3^ About 417 754 hectares in India are used for tobacco cultivation producing about 749 907 tons of unmanufactured tobacco^3^ as of 2018. The gross value added (GVA) from tobacco manufacturing was INR287.96 billion in 2018–2019 which constituted about 1% of the total manufacturing GVA.^4^ Employment in tobacco sector activities is estimated at 7.25 million.^5^ Employment in tobacco manufacturing constituted only 0.2% of rural and 0.1% of urban male population (ages 15–59) and 1.1% of rural and 0.8% of urban female population in the same age group in 2-11-12.^6^ Total excise tax revenues from tobacco amounted to INR217.19 billion in 2016–2017. However, it was about 5.8% of central excise tax revenues and only 1.3% of total gross tax revenues.^7^

Notwithstanding the contribution of tobacco sector to the national economy, tobacco use generates huge economic burdens on society. In India, about 930 000 people die annually on account of smoking attributable diseases^8^ and 350 000 die on account of smokeless tobacco (SLT) use attributable diseases,^9^ resulting in 3500 tobacco-attributable deaths per day. The economic burden associated with tobacco use is estimated to be Rs.1773.4 billion (1% of India’s gross domestic product (GDP)) in 2017–2018, 74% due to tobacco smoking and 26% to SLT use.^10^

In 2017, India overhauled its indirect tax system with the implementation of the goods and services tax (GST), which subsumed federal-level excise duties and state-level value-added taxes. However, in the 7 years since its introduction, tax rates on tobacco products have remained largely unchanged, increasing its affordability.^11^ The tax share in the final retail price remains below the WHO-recommended 75%,^12^ with approximately 51% for cigarettes, 22% for bidis and 64% for SLT.^11^ Although taxation is recognised as one of the most cost-effective tools to regulate tobacco use,^12 13^ it appears that tax policy is underused in India.

It is important that policies to regulate tobacco products such as taxation assess their net impact and examine whether it would lead to a net benefit to the economy and society at large. For example, a tobacco tax increase, apart from its direct impact on reducing consumption, can have indirect effects on consumption and production of various other products in the economy. For example, land and labour released from tobacco sector may be used in other sectors. Thus, it is important to examine the tobacco sector as part of the whole economy to better understand the economy-wide impact of tobacco control policies. The National Health Policy (NHP) 2017^14^ envisages a 30% relative reduction in current tobacco use by 2025. While this is desirable from a public health perspective, there is a need to examine how such a reduction in tobacco consumption would impact rest of the economy, for example, via the flow of all economic transactions that take place.

The literature on the net impact of different tobacco control policies after allowing for multisectoral linkages has been limited. A study^15^ in the UK found a net increase in employment when there was a decrease in tobacco consumption and spending. Input–output analysis was used in a study from Canada^16^ which found significant reallocation effects following a consumption decline resulting from tobacco control legislation. Employment effects were found to be more pronounced in the public sector than in the private. Multisector computable general equilibrium (CGE) modelling study in Taiwan^17^ found a new tax scheme would significantly reduce cigarette consumption and result in significant gains in health benefits. Using IO model, a study^18^ from Indonesia estimated that a 100% tax scenario would be positive in terms of output produced by all sectors. A study from Bangladesh^19^ found that reducing tobacco consumption and redirecting the decreased demand as a stimulus to other sectors of the economy led to a significant increase in GDP and national income. A CGE modelling study from Tanzania^20^ estimated that a reduction in tobacco consumption would reduce employment in the tobacco sector but increase employment in other sectors. The overall decline in total economic output was minimal, estimated at −0.3%. Another study^21^ from Vietnam used Input-Output (IO) tables to find that the net impact on the national output and employment would be positive for any increase in the excise tax on tobacco. An Argentine study^22^ using CGE found a 15% rise in tobacco taxes could lead to a shift in jobs from tobacco-related sectors to other sectors in the economy. The overall impact on total employment was negligible, while health outcomes improved. A study from Pakistan^23^ used CGE modelling to estimate the impact of increasing the tax burden on cigarettes to 70% of the retail price. This would result in an overall employment income increase of 0.5% in the economy. Additionally, it projected that overall household income, GVA and output would increase by 0.13%, 0.12% and 0.03%, respectively. A study from Mexico^24^ used an applied general equilibrium model to examine the impact of increasing the specific component of the Excise Tax on Production and Services to 76% of the retail price. Findings suggest minimal loss of employment in the tobacco industry would be more than offset by job creation in the health sector.

There are only a few studies in India which examined the impact of different tobacco control policies on various outcomes of interest. One found the introduction of GST would make tobacco tax structure more complex and most states would experience a fall in tobacco tax revenue.^25^ A cigarette tax increase of Rs.10 plus 10% ad valorem would lead to 1.5 million men quitting smoking, averting 665 000 deaths, and generating additional tax revenue of about Rs.4385 crores among other benefits.^26^ A recent study examining the impact of the Government’s National Tobacco Control Programme concluded that early implementation of the programme may not have led to reductions in tobacco use.^27^ None of the studies, however, examined the multisectoral impact of these policies. This study fills that gap using a CGE model.

## Methods and data

CGE is a framework in which the linkages between various sectors and the allocation of endowments/resources (such as land, labour and capital) are captured. This framework accounts for the fact that such resources are fixed in the short-to-medium term while sectors can expand and contract, depending on how much their production is needed by other sectors and by final consumers. The current study developed a full-fledged global multisectoral multiregional CGE model, which has comprehensive details on tobacco use and tobacco products/sectors. This is based on the Global Trade Analysis Project (GTAP) model and database,^28^ which includes data on aggregated sectors such as crops and beverages/tobacco products. A detailed description and updated technical documentation on GTAP are available.^29 30^ Moreover, a detailed explanation of the CGE model, the GTAP database, including its baseline and scenario design, is available in online supplemental appendix. For the purpose of more disaggregated product-wise analysis, we further augmented GTAP model with country-specific information on tobacco crop, tobacco products such as bidis, cigarettes and SLT, employment in tobacco sector, tobacco tax revenue, own-price and cross-price elasticities of tobacco products, and economic burden from tobacco use in India from a variety of secondary sources including India’s IO table^31^ with information on raw tobacco and tobacco product sectors.

The tobacco control policy scenario we considered for the CGE simulations was a uniform 10% reduction in consumption of bidis, cigarettes and SLT by 2026. The health benefits from this policy scenario in terms of number of deaths averted and consequent benefits to the economy coming from increased labour supply were also estimated in an extension to the CGE framework. The model achieved the targeted reductions in consumption through either a tax on raw tobacco or a tax on consumption. The former reflects the possibility that taxing at an earlier point in the supply-chain might increase compliance, particularly for informally produced goods like bidis and SLT. Although the model in this paper is time-agnostic for the simulation of consumption reduction, we assume the consumption reduction to happen over a 5-year period starting from the time of policy intervention. We used the own-price elasticities of −0.91 to –0.35 and −0.88 for bidi, cigarette and raw tobacco leaves, respectively, from existing literature.^32^ Similarly, the income elasticity for bidi, cigarette and tobacco leaves is 0.39, 1.98 and 0.33, respectively. These values represent the average income elasticities for these products in rural and urban India.^33^

While the model simulates the resulting changes in production, consumption and employment the resulting impact in terms of deaths averted were estimated outside of the model. We first computed a number of current tobacco users and estimated the number of deaths at baseline assuming 33% of all users die prematurely, a very conservative assumption based on the existing literature.^34^ We also used an alternative, less conservative scenario, assuming 50% premature deaths among tobacco users. On this 33%, we applied a mortality adjustment factor for 67%. It means, only 67% of the 33% premature deaths would be averted if they quit tobacco use as in existing literature.^34^ We multiplied these estimated averted deaths with the labour force participation rate among the adult population (48.53%) for 2021 as obtained from the World Bank. The gain in labour force thus estimated was plugged back into the CGE model as a shock to the labour force in the model. Given that the estimated deaths averted are for the remaining life course of those quitting, it is important to annualise it to estimate the number of averted deaths which are potentially part of the labour force. For this, we first estimated the difference between average age of tobacco users under 65 years (39 years for SLT users and 42 years for smokers as estimated from GATS) and the age of being out of the labour force, assuming 65 years. An additional 2.5 years was added to this difference as the quitting is assumed to happen uniformly over the 5-year period during the consumption reduction. The total averted deaths are then divided by this number to estimate an annual number of averted deaths among those in labour force and apply for this number as a shock to the labour force in the CGE model to derive the resulting change in GDP for the policy shock scenarios over the 5 years.

## Results

Table 1 shows the size of tobacco sector in Indian economy as used for the macroeconomic simulation in the model. The combined contribution of bidis, cigarettes and SLT in the value of total production in Indian economy was 0.32% while that in the value of consumption was 1.05%. The combined contribution to the employment was at 2.2% and the sector’s total share in taxes was at 0.99%. More than 87% of the employment contribution from tobacco sector came from the bidi sector while 91% of the tax contribution in tobacco sector was from cigarettes.

**Table 1:** Baseline (2021) information on different tobacco products in the model: (in millions of USD unless specified)

	Bidis	Cigarettes	Smokeless tobacco	Tobacco sector	Overall economy
Production	4416.96	14 320.19	1475.99	20 213.14	6 263 965
Consumption	4744.93	14 518.54	1263.71	20 527.17	1 960 253
Employment (millions of jobs)	8.93	0.94	0.35	10.22	471
Export	6.51	97.59	288.04	392.14	531 092
Import	0.01	19.41	10.30	29.72	633 277
Tax revenue	427.04	5517.05	113.73	6057.82	609 988
Contribution to production (%)	0.07	0.23	0.02	0.32	100
Contribution to consumption (%)	0.24	0.74	0.06	1.05	100
Tax burden (%)	22	49.5	63.75		

Table 2 shows the macroeconomic impacts of the consumption reduction on the key outcome variables. The target reduction in consumption is achieved either through a tax on consumption (scenario 1) or a tax on raw tobacco (scenario 2). The tax on consumption results in a 0.14% reduction in GDP and 0.44% reduction in overall employment. On the other hand, tax on tobacco would lead to a GDP reduction of 0.12% and employment reduction of 0.42%. Both GDP and employment impact appears to be similar irrespective of the method used to reach the targeted consumption reduction. However, tax increase is higher when the tax is imposed on tobacco consumption. While a tax on tobacco consumption generates an additional tax revenue of US$2774 million, tax on raw tobacco generates and additional tax revenue of US$2142 million both achieving the same targeted reduction in tobacco consumption. While the scenarios 1 and 2 increase tax, the actual increase of tax from tobacco sector was higher than the net increase. Cigarette is a major contributor to tax revenue because bringing down cigarette consumption requires a relatively higher tax imposition. This is because while the tax increases in the tobacco sector there is a simultaneous reduction in tax from other sectors due to changes in consumption that occurs in other sectors.

**Table 2:** Changes in outcome variables in the overall economy due to a targeted reduction in consumption of bidis, cigarette and SLT by 10% by year 2026

	Taxing tobacco consumption	Taxing raw tobacco
GDP change (%)	−0.14	−0.12
GDP change (millions USD)	−4456.34	−3738.41
Production change (%)	−0.15	−0.11
Production change (millions USD)	−9132.20	−6819.17
Change in consumption (%)	−0.19	−0.14
Change in consumption (million USD)	−4437.87	−3288.38
Private consumption (%)	−0.23	−0.16
Private consumption (million USD)	−4533.78	−3171.20
Government consumption (%)	−0.03	−0.02
Government consumption (million USD)	−100.12	−65.22
Export (% change)	0.43	0.19
Exports change (millions USD)	2293.97	1030.37
Import (% change)	−0.21	−0.16
Imports change (millions USD)	−1309.93	−1030.45
Employment (% change)	−0.44	−0.42
Employment change (million jobs)	−2.07	−1.97
Tax revenue (% change)	0.79	0.61
Tax revenue (change in millions of USD)	2773.82	2141.81
Change in tax from tobacco sector	3051.21	1770.09
Change in tax from non-tobacco sector	−277.38	−371.72

GDP, gross domestic product ; SLT, smokeless tobacco.

Table 3 shows the impact that alternative methods of achieving the target consumption reduction have on tobacco consumption and production. In the first scenario where we reduce the final consumption of bidi, cigarettes and SLT, there is a decline in production volume and production value. On the other hand, in the second scenario, where we tax the raw tobacco, there is a decline in production volume, but an increase in the production value. In the first scenario, we are shocking the tobacco consumption value after taxes, the production price will not increase and so both production volume and value declines. On the other hand, in the second scenario, where we tax raw tobacco, the production value will increase as the price now also includes this new tax. So, the production value increases in scenario 2. Unlike production value, the consumption value is the value after taxes. So, when we shock the consumption volume, it drives the consumption prices increasing the consumption value in both scenarios. Also, it must be noted that the consumption value of cigarettes is higher than bidi and SLT in both scenarios. This is due to the own price elasticity of cigarette consumption which is much smaller in magnitude compared with both bidis and SLT. It means a given percentage increase in prices (due to a change in tax) will have a relatively smaller impact on consumption of cigarettes than on the consumption of bidis and smokeless. So, the consumption price should increase at a higher extent than for bidi and SLT to achieve the same reduction in consumption. Considering production value of cigarettes, a similar trend prevails and it is higher than the increase estimated for bidis and cigarettes. This is because we tax raw tobacco way before the production stage and so the production price of cigarette should increase to achieve the consumption decline whereas in the first scenario, the production value declines because we are only shocking the final consumption and the production prices may not increase.

**Table 3:** Impact on consumption and production in tobacco sectors under alternative policy implementation routes

	Bidis	Cigarettes	Smokeless
Change in consumption (value)			
Base value (million USD)	4744.93	14 518.54	1263.71
Taxing tobacco consumption	3.58%	11.39%	3.78%
Taxing raw tobacco	3.58%	11.39%	3.78%
Change in consumption (volume)			
Taxing tobacco consumption	−10.00%	−10.00%	−10.00%
Taxing raw tobacco	−10.00%	−10.00%	−10.00%
Change in production (value)			
Base value (million USD)	4416.96	14 320.19	1475.99
Taxing tobacco consumption	−8.39%	−7.73%	−6.79%
Taxing raw tobacco	1.96%	3.14%	1.64%
Change in production (volume)			
Taxing tobacco consumption	−8.32%	−7.72%	−6.66%
Taxing raw tobacco	−9.27%	−10.33%	−7.34%
Marginal increase in tax burden			
Taxing tobacco consumption	13.69%	21.50%	13.82%
Taxing raw tobacco	15.26%	30.25%	15.24%

All baseline numbers in this model are in millions of USD. Volume change is the value change in constant prices. Therefore, volume changes shown here are the same as value changes in constant prices.

The direction of change can be different for values of consumption and production as against the volume of consumption/production. This is because, even if the real consumption volumes decrease over the 5-year period, their monetary values could still turn out to be positive due to increased (inflation-adjusted) prices over the 5-year period. The total tax burden increases for all three tobacco products under both policy implementation routes compared with the baseline tax burden.

Table 4 shows the sectoral impact on employment without accounting for the additional health gains from tobacco use consumption reduction. As we can see, the target consumption reduction would lead to a loss in employment in bidi, cigarettes and SLT. There will be about 8.4%–9.3% decline in employment in the bidi sector full-time (approximately 640 000–709 000 job loss over the 5-year period) depending on the scenario chosen. Similarly, the loss of employment in cigarettes sector would be 8%–8.4% (approximately 74 000–78 000 job loss over the 5-year period) and for SLT would be 6.8%–7.6% (approximately 23 000– 26 000 job loss over the 5-year period). There is net growth and decline in employment in other sectors too. The overall impact on employment, without accounting for the health gains from consumption reduction, for all sectors combined was, negative, although low at 0.44% (approximately 2.06 million job loss over the 5-year period), as discussed in table 2.

**Table 4:** Sectoral impact* on employment due to a targeted reduction in consumption of bidis, cigarettes and SLT by 10% by year 2026

Sectors	Baseline jobs (millions)	Taxing tobacco consumption	Taxing raw tobacco
Bidi	8.94	−8.425	−9.347
Cigarette	0.94	−7.974	−8.372
Smokeless	0.35	−6.785	−7.569
Sugar cane, sugar beet	11.24	−0.657	−0.514
Sugar	0.75	−0.604	−0.523
Trade	30.34	−0.512	−0.393
Mineral products nec	1.95	−0.488	−0.384
Accommodation, food and services	6.21	−0.488	−0.323
Dwellings	0.20	−0.475	−0.312
Construction	46.26	−0.473	−0.365

* Only 10 sectors each with the highest and lowest decline in employment are shown.

SLT, smokeless tobacco.

The simulation estimates that 3.08 million deaths were averted due to a target 10% reduction in consumption. Because the estimate of averted deaths is based on the percentage reduction in consumption, the estimated number of averted deaths was the same whether we use scenario 1 or scenario 2 to achieve the consumption reduction. Table 5 shows what happens to the GDP if we are able to incorporate a fraction (48.53%, which is the labour force participation rate) of the averted deaths or saved lives resulting in consumption reduction as an increase in the available supply of labour force. As we can see, the gain in GDP over the 5-year period after accounting for averted deaths, and under the more conservative assumption on premature deaths among tobacco users, was positive at 0.36% which, in absolute terms, was US$11.43 billion and net gain was positive at 0.22%, which, in absolute terms, was US$6.99 billion. This net gain is the sum of GDP loss resulting from the original scenario (0.14%) and gain in GDP arising from accounting for the addition of saved lives into the labour force. Since the labour force participation rate was 49.5%, the increase in labour force was 1.2 million. One can see that the net gain is positive and is achieved within 5-year duration of the simulation itself. Since the overall impact on the employment was a loss of 2.06 million jobs over 5 years before accounting for the health gains, the net change in employment would be a gain of 1.36 million jobs over the 5 years term. If on the other hand, we used a relatively less conservative assumption of 50% tobacco users dying prematurely, we can see the net impact on GDP is even larger at 0.37% translates to US$11.76 billion and a net gain in employment for 1.51 million jobs over the 5-year term.

**Table 5:** Impact on GDP over 5 years due to additional health gains from the policy shock

	Rows	Outcomes with alternative assumption on premature deaths from tobacco use	
		33% users dying prematurely	50% users dying prematurely
Total change in GDP without assuming any lives saved (%)	(A)	−0.142	−0.142
Deaths averted over the 5-year period (‘000)	(B)	3080.03	3696.04
Increase in labour force due to deaths averted (%)	(C)	0.730	0.84
Gross increase in GDP after accounting for averted deaths (%)	(D)	0.363	0.514
Net increase in GDP due to the policy shock after accounting for averted deaths (%)	(A+D=E)	0.221	0.372

GDP, gross domestic product.

## Discussion

Recognising the harm caused by tobacco use, the NHP 2017, set out to achieve a 30% relative reduction in consumption of ‘current’ tobacco use by 2025. However, given the interlinkages of tobacco sector with the other sectors in Indian economy, it is important to examine whether various policy actions on tobacco control would lead to a net benefit to the country. This paper uses a CGE model to determine the macroeconomic impact of a targeted 10% relative reduction in consumption of bidis, cigarettes and SLT seeking to achieve these either through a tax on consumption of tobacco or a tax on the raw tobacco. To achieve a 10% consumption reduction, excise taxes would need to be increased by 10.98% for bidis, 28.57% for cigarettes and 30.30% for SLT. The results indicate that such a reduction in the consumption of all three tobacco products results in a 0.14% reduction in the GDP and 0.44% reduction in overall employment in the economy. Once we account for the gains in employment as a result of an increase in the number of deaths averted due to reduction in tobacco use consumption, it results in a net 0.22% increase in GDP and 1.36 million net increases in employment over the 5 years. The same will result in a 0.37% increase in GDP and 1.51 million net increases in employment over the 5 years if we take a slightly less conservative approach of assuming 50% of all tobacco users die prematurely. This study underscores the benefits of actively pursuing fiscal policies to control tobacco use in India, with positive outcomes in terms of net increases in GDP as well as overall employment in the country. Its findings are consistent with similar results we reviewed earlier from other countries. It is also important to note that the model is time agnostic. So, the effects estimated in the model are assumed to be valid for the 5 years from the time the policy changes come into effect irrespective of the assumed baseline.

While this study applies a novel approach to the analysis of the economy-wide impact of targeted reduction in consumption of tobacco products in India, the study has certain limitations. First, the model examines a concurrent increase in tax burden across all three tobacco products or an increase in tax on all raw tobacco depending on the scenario chosen for the target consumption reduction. If taxes are not increased on any one of the products as has been done in case of bidis in India post the introduction of GST, the results may not hold. The model rather highlights the need for a simultaneous tax increase on all tobacco products. Second, the model assumes that tax increases have a 100% pass through effect and increase the prices by the same rate. Although it is not necessary, it has been observed that tobacco industry, often, increase prices by more than the increases in tax. In such cases, the consumption impact estimated in the model is understated. Third, our estimates of deaths averted and the subsequent positive shock on the labour force are conservative since we used a very conservative assumption that only 33% of all tobacco users die prematurely compared with many studies assuming 50%. Fourth, this study incorporates the health gains only in the form of averted deaths into the CGE modelling framework to examine its impact on GDP. However, reduced disabilities, morbidities and increased productivity on account of reduction in tobacco use can have an additional impact in terms of improving labour productivity which will also positively impact GDP. This study did not fully explore how the allocation of extra government revenues from tobacco taxation towards growth-enhancing programmes, like health and education, might improve growth prospects. To this extent, the gains in GDP as estimated in this report are rather conservative. Fifth, in the absence of estimates of consumption elasticities for different tobacco products in India, the model makes a simple assumption that prevalence elasticity constitutes half of the own-price elasticity as per available evidence from the international literature. Sixth, the model achieves the target reduction in consumption through a certain increase in tax burden either on consumption of tobacco products or on the row tobacco. It does not examine the impact of changing different components of taxes. In India, the tax on tobacco has different elements including excise tax, GST and compensation cess.^25^ A given percentage increase in each of these components will have different effects on the final price and tax burden. Hence, the tax increases warranted will differ according to the particular component of the tax that is increased in order to get the required tax increase for the target reduction in consumption. Finally, we assume standard neoclassical economic tenets like perfect competition, constant returns to scale and specific (although highly flexible) functional forms, which may or may not hold true in reality for tobacco and other sectors. Notwithstanding these caveats, the CGE modelling offers several insights into the potential tobacco control policies that India could adopt and their relative outcome in terms of changes in employment, output and GDP.

## Conclusion

Tobacco industry has always exaggerated the importance of tobacco sector in the Indian economy, sometimes even with the help of published, non-transparent, reports.^35^ However, it has never considered the positive gains from tobacco control policies and the resulting health gains at the macroeconomic level. This study finds that the macroeconomic impact of target consumption reduction of tobacco achieved through tax increases either on the consumption of final tobacco product or on the raw tobacco is positive and will increase GDP once health gains from reduced consumption are taken into account. Due to the lack of the major increase in taxes on tobacco products since the time GST was introduced in India in 2017, except for marginal increases in 2021 and 2023, tobacco products have become increasingly more affordable over the past few years.^36^ For the government of India to stay on its target of achieving significant reductions in tobacco use consumption, it is imperative that tobacco taxes are increased more significantly and simultaneously for all tobacco products and on a regular basis. As this study shows, such tax increases will be a win-win for the country considering its positive macroeconomic impacts over time resulting from net increases in GDP as well as overall employment.

## Supplementary material

10.1136/tc-2023-058471 online supplemental file 1

## Data Availability

No data are available.
