# Supplementary material for: Economy-wide impact of a reduction in tobacco use in India
Source: Tob Control. 2024 Sep 27;34(6):e058471. doi: 10.1136/tc-2023-058471 (PMC12703278; doi:10.1136/tc-2023-058471)
Supplement: online supplemental file 1 [file tc-34-6-s001.pdf]

Economy-wide impact of a reduction in tobacco use in India

Rijo M. John, Badri Narayanan Gopalakrishnan, Sumathi Chakravarthy, Sindhu Bharathi M., Praveen Sinha, Vineet Gill Munish, Mark Goodchild

APPENDIX

COMPUTABLE GENERAL EQUILIBRIUM MODEL

Computable General Equilibrium (CGE) models have been widely used by the global research community to answer pressing policy questions. In a nutshell, the CGE model is a framework in which the linkages between various sectors and the allocation of endowments/resources (such as land, labor and capital) are captured. This framework accounts for the fact that resources are fixed in the economy while sectors can expand and contract, depending on how much their production is needed by other sectors and by final consumers. For each sector, a typical CGE dataset comprises the inputs needed for production, in terms of factors as well as materials used from production of other sectors, imports, and details of where the output goes: domestic or exports. What happens in one sector has the potential to affect the whole economy, not only through its own share in the economy but also through its forward and backward linkages with other sectors. In other words, tobacco may be a small sector vis-à-vis the size of the Indian economy, but if we account for its linkages with other sectors in the economy, it can be very significant.

Starting from the premise that consumption is reduced by X% in N years, a Partial Equilibrium (PE) model approach would translate such reduction in terms of change in aggregate consumption of tobacco in N years, by accounting for per-capita income growth and elasticity of tobacco consumption with respect to income and prices. Such a model captures the supply-demand linkages in the tobacco sector, by accounting for production, consumption, exports and imports in raw and processed tobacco. There are behavioural equations in production, private consumption, exports and imports, as well as market clearing conditions to equate supply with demand. These are based on data on different variables outlined here as well as elasticities some of which were estimated from data and some of which were taken from the literature. Employment effects are then captured by assuming a simple multiplier on output.

This is too simple an approach and has the limitations of not being comprehensive enough in an economy-wide sense, and also even within tobacco markets, since it ignores the intermediate

inputs involved in production of tobacco and its products. Further, effects on other sectors as well as broad macro-economic effects are also not captured in this model. It also ignores international effects, emanating from the rest of the world.

To address the limitations above, we develop a full-fledged global Computable General Equilibrium model, which has comprehensive details on tobacco and tobacco products sectors. This is based on the GTAP model (Hertel, 1997) and database, further augmented significantly using country-specific data on tobacco crop and tobacco products, from various national and international sources of data as explained in our report.

Such a model captures supply-chain effects, macro-economic aspects, economy-wide equilibrium constraints, linkages between different sectors and countries, as well as emission and land use effects of different commodities. We can also capture the potential substitution of tobacco for other crops in the case of the former's decline. We shall model the tobacco control policy in terms of reduction in production and trade of this sector.

For the CGE analysis in the study, we developed a full-fledged global multi-sectoral multi-regional CGE model, which has comprehensive details on tobacco and tobacco products/sectors. This is based on the Global Trade Analysis Project (GTAP) model and database[34], which includes data on aggregated sectors such as crops and beverages/tobacco products. For the purpose of more disaggregated product-wise analysis—separate analysis on cigarettes, bidis and smokeless tobacco—we further augmented the GTAP model with several country-specific information on tobacco crop, tobacco products, employment in the tobacco sector, tobacco tax revenue, own-and cross-price elasticities of tobacco products, economic burden from tobacco use etc. in India from a variety of published secondary sources.

Figures A1 and A2 illustrate some key aspects of the quantity flows and the price transmission channels in the model, respectively.[1] The model developed in this paper is an extension of the standard GTAP model (Global Trade Analysis Project, 2018). We define the sets SECT of sectors (indexed by k) and REG of regions (indexed by r in most cases and if the region is the source of exports/imports but by s if the region is destination of exports/imports). We turn now to a discussion of key components of the GTAP model.

[1] The unit of measurement for all variables explained in this section is percentage change.

International Trade

The change in imports of each region from each of the others is determined by three factors: (i) substitution among different sources, based on the differential between import prices from specific sources and the sum of import-augmented technical change and aggregate import prices $pimk_{k,s}$[2], multiplied by the elasticity of substitution of imports between the sources $\sigma_{M,k}$, which is the Armington elasticity for the sector as in GTAP Database, (ii) import-augmenting technical change, $amsk_{k,r,s}$, that lowers the effective price of a good in the destination market, and (iii) the import penetration as captured by change in composite imports of subsector commodity *k*, $qimk_{k,s}$: :

For all sectors k in SECT, regions r and s in REG:

$$qxsk_{k,r,s} = -amsk_{k,r,s} + qimk_{k,s} - \sigma_{M,k} * [pmsk_{k,r,s} - amsk_{k,r,s} - pimk_{k,s}] \quad (1)$$

Global transport margins are treated in the same manner as in the standard GTAP model, with the quantity of international trade, transport and insurance services required being a fixed proportion of the volume of goods shipped. Technical change in this sector is represented with the variable $atmfsdk_{k,r,s}$ is obtained by adding up the changes at different levels, which are directly translated from the aggregate changes in the corresponding variables. Trade and transport services are provided at a common price, *pt*, which represents a Cobb-Douglas aggregation of trade and transport services exports from all regions in the model. Deducting the rate of technical progress from this price change gives the percentage change in the commodity and route-specific transport margin, $ptransk_{k,r,s}$. The price linkages in the model also include export taxes $txsk_{k,r,s}$, export *fob* prices $pfobk_{k,r,s}$, and import *cif* prices $pcifk_{k,r,s}$ as shown in Figure A2. Changes in import tariff and export taxes are the policy variables here.

Domestic Consumption

There are three broad categories of consumption of products and services manufactured in a country: private households, government and firms. In addition, each of these categories of agents also consume imports that are aggregated across exporters, based on the descriptions in section a above. For private households, GTAP assumes CDE (Constant Difference Elasticity) functional form, which is flexible enough to have Linearized Expenditure Systems (LES) and Constant Elasticity of Substitution (CES) as special cases. For government to consume different products and for firms to

[2] The substitution effect for a particular flow *(k,r,s)* increases in divergence of import tariff for good *k* from regions *r* to *s*, from the weighted-average tariff of *s*. Since higher weight means lower divergence, this effect decreases in import-shares of region *r* in the total imports by region *s* of the good *k*.

consume different intermediate inputs, the functional form is CES. There is also a CES nest between domestic and imported products for each of these agent.

Domestic Production

Production function in GTAP involves 3 levels of nests: (1) There is a Leontief function on the topmost part of production system, wherein intermediate inputs as a composite single input and primary factors as another composite single input are complements. (2) Within the intermediate inputs, there is a CES function. (3) Within the primary factor inputs, there is a CES function. With the exception of land and natural resources, which can move only within agricultural and extraction sectors respectively, other factors are mobile across sectors. GTAP-E model, which focuses on energy and environmental aspects, modifies the system above to introduce substitution between energy sectors and capital input, while further introducing substitution (CES) between different types of energy sources to capture channels of emissions reduction.

Links between Production, Consumption and International Trade

The sub-modules explained above are linked with each other. The percentage change in sector-level domestic consumption, $qdmk_{k,s}$, with corresponding price change $pmk_{k,s}$, substitutes for imported subsector goods, $qimk_{k,s}$, with corresponding price change $pimk_{k,s}$. The CES elasticity between these two variables is $\sigma_{D,k}$, and this substitution takes place based on their respective price differentials from the sector-level domestic prices $pdk_{k,s}$, as illustrated by equations (2) and (3):

For all *k* in *SECT* and *s in* REG:

$$qimk(k,s) = qdk(k,s) - \sigma_D(k) * [pimk(k,s) - pdk(k,s)] \quad (2)$$

$$qdmk(k,s) = qdk(k,s) - \sigma_D(k) * [pmk(k,s) - pdk(k,s)] \quad (3)$$

Domestic market and import price changes are aggregated to domestic price changes by weighting according to their respective shares. $VDK_{k,r}$ is the total value of domestic consumption of goods corresponding to the sub-sector *k* in the region *r*, $VDMK_{k,r}$ is the value of domestic consumption of goods produced by the domestic sector *k* in the region *r* and $VIMK_{k,s}$ is the value of imports of goods produced by the sub-sector *k* to the region *s*.

For all *k* in *SECT* and *s in* REG:

$$pdk_{k,s} = \alpha D_{k,s} * pmk_{k,s} + \alpha M_{k,s} * pimk_{k,s} \quad (4)$$

where: $\alpha D_{k,s} = VDMK_{k,s} / VDK_{k,s}$ and $\alpha M_{k,s} = VIMK_{k,s} / VDK_{k,s}$

Finally, the total changes in supply and demand are equalized to ensure equilibrium, by equating the percentage change in total output $qok_{k,r}$ with the share-weighted sum of exports and domestic

consumption for all sectors k in SECT and regions r in REG. When the slack variable $tradslackk_{k,r}$, is exogenized, this equilibrium condition determines the change in market prices, $pmk_{k,r}$ (output, $qok_{k,r}$, is determined by Equation (5).

For all *k* in *SECT* and *r in* REG:

$$\mathbf{qok}_{k,r} = \beta D_{k,r} * \mathbf{qdmk}_{k,r} + \Sigma_s \beta M_{k,r,s} * \mathbf{qxsk}_{k,r,s} + \mathbf{tradslackk}_{k,r} \qquad (5)$$

Figure A1: Diagrammatic Illustration of the Quantity Flows in the Model[3]

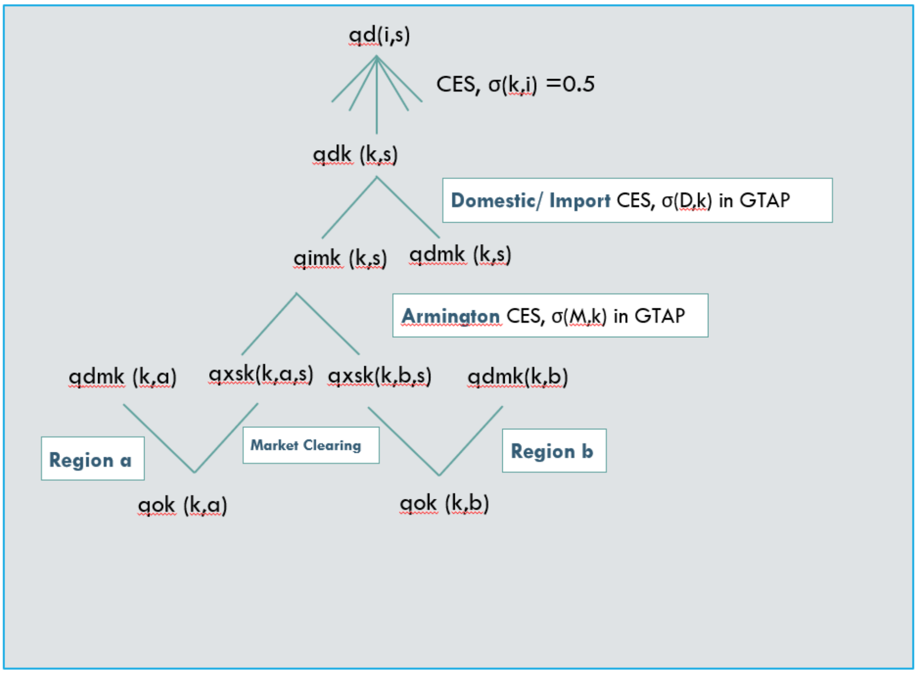

Figure A2: Illustration of Price Linkages in the Model

[3] Notations in Figures A1 and A2 follow standard GTAP notations[46]. In general, the variables starting with: '*q*' represent changes in quantities, '*t*' represent tax/tariff changes and '*p*' represent changes in prices. Those ending with '*k*' are at disaggregate level. In the variable-names, '*d*' stands for domestic, '*i*' for imports, '*x*' for exports, and '*o*' for output. Indices '*r*' and '*s*' denote source and destination regions, respectively; '*k*' denotes sectors.

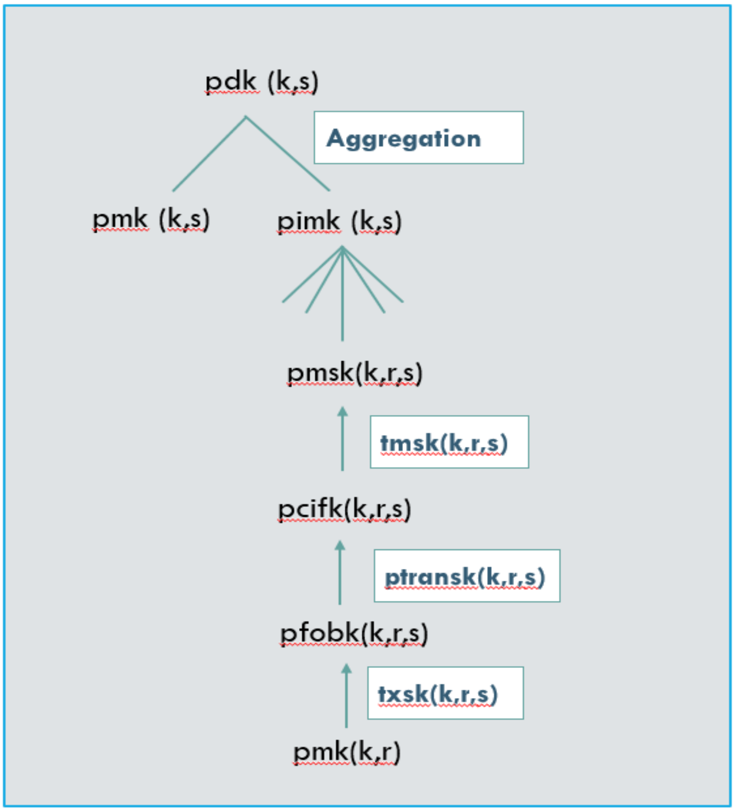

Figure A3 provides a simplified view of the accounting structure of GTAP model. Regional household receives all the income from tax revenue (TAXES) and factor payments (VOA), from the different agents in the economy, namely private households (PRIVEXP), producers or firms and government (GOVEXP). After spending all the revenues for private consumption and government consumption (based on a Cobb-Douglas Utility function that largely preserves the initial shares of PRIVEXP and GOVEXP in total regional income), the residual is savings.

Savings is then accumulated by 'Global Trust' across the world, and then distributed to different regions as investment (REGINV), based on their performance in terms of rates of returns. This is then added to the productive capital of the firms, which uses the different factors (VOA) and intermediate inputs – both domestic (VDFA) and imported (VIFA), to produce output. This output is then exhausted between private households (VDPA), government (VDGA) and also intermediate inputs across different firm types (VDFA). Of course, the households can also import (VIPA), and so can the governments (VIGA).

In Figure A3, the international transactions (with Rest of the World) are shown in red, while the domestic ones are shown in blue. Price linkages happen through this accounting framework; for example, the percent change in prices paid by the exporter before shipping the commodity

to other countries are merely a sum of the percent change in market prices and export taxes (XTAX), while that in prices paid by the importer would include both the transportation cost and import tariffs (MTAX), on top of this export price. Investment in this model is governed by a mechanism of diminishing marginal returns to capital and recursive dynamics that captures adaptive expectations with error corrections in expected rates of return.

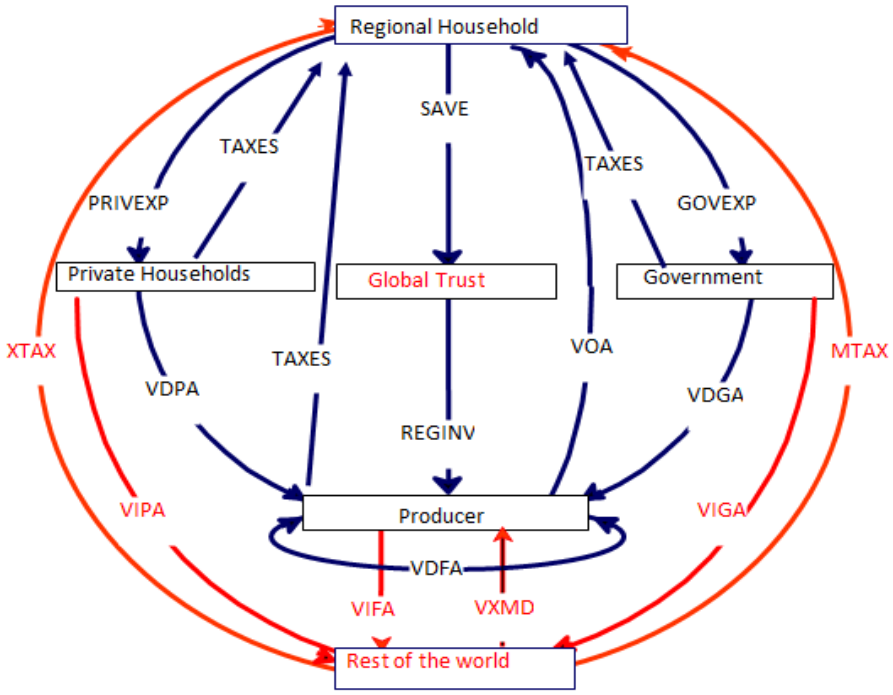

Figure A3: Simplified view of the Accounting Structure of the GTAP model

This model (GTAP) has been widely employed both in the academic and policy world to understand the ex-ante economic impacts of global shocks under different scenarios. At the risk of simplification, the simulations generated from the model offer a picture of how the economies would look when different shocks are administered. These models offer a convenient way to represent all the agents in an economy and their complex interactions with each other, thereby capturing the general equilibrium effects in the form of a mathematical system. When an economy in equilibrium is disturbed by shocks, how do those effects play out and what is the direction and magnitude of such effects? It goes without saying that the

reliability of the results are entirely dependent on the fundamental assumptions made in the scenarios.

GTAP DATA BASE EXPLANATION

This appendix overviews the GTAP data construction, and how the data from the different sources are reconciled together. Data collected by one country is rarely consistent with data collected by another due to inaccuracies in the collection and compiling of data. An example of this is in the trade data, where the amount or value of exports one country claims to sell to another rarely matches what the other country claims to import or buy from them, even after transportation and other costs are taken into account. Even within the same country, the same data collected from two alternative sources may differ due to differences in how the data is being collected, interpreted, classified, and valued, notwithstanding the differences caused by errors and omissions. For example, GDP collected from the expenditure side never equals GDP collected from the production side, there is always a statistical discrepancy.

However, global economic analysis requires consistent and reconciled global data. The reconciliation of global datasets is laden with judgments about the quality of the alternative data sets being reconciled. In the case of the GTAP Database the externally collected trade, macroeconomic, ITC protection and IEA energy data are contributed based on international sources and are considered superior to the individual country data because they have gone through this standardization and reconciliation process, although little research has been undertaken to compare the sources.

Economic data for each country including the value of inputs and uses of production is provided as an IO table by a global network of contributors. The starting point for most contributors is an I-O table, supply and use tables, or social accounting matrix developed by the country's statistical office. Most tables require revisions to be made by the contributor to get the original table into a format ready for GTAP. Several constraints need to be satisfied by these IO tables, particularly in terms of signs, balance and other aspects such as treatment of re-exports, domestic/import splits, etc.

The contributed input-output tables which have already satisfied the guidelines for contributors, then go through a cleaning procedure and are pre-processed to produce consistent 65 sector tables. This pre-processing includes minor cleaning, disaggregation, development of

IO tables for composite regions and targeting the IO tables to the international datasets for agricultural production (OECD and FAO).

Those I-O tables which do not have full agricultural and/or non-agricultural disaggregation then go through the I-O disaggregation procedure. Agricultural IO tables based on FAO data are used to disaggregate agriculture. This ensures that the cost shares of the disaggregated agricultural commodities look reasonable. Non-agricultural disaggregation is done using the shares obtained from a 'representative table', which is generated as a GDP-weighted average of all contributed IO tables that have all 57 sectors to begin with. Disaggregation does not alter the aggregated totals; it merely apportions the cost and use structures while keeping the same totals.

Input-output tables are constructed for all composite regions – GTAP regions for which there are no contributed tables. This is done by matching each country within the composite region with a country for which we have an IO table. This matching is done using entropy method, by identifying the 'match' in terms of per-capita GDP within the same geographical region. This country's IO table then act as a proxy for the missing country. Later all the proxy IO tables for countries in each composite region are aggregated to form the composite regions. At this stage of data base construction, all countries have a harmonized set of 65 sectors, which can be added up to the original contributed IO tables for each country.

A single procedure is used to achieve the next three objectives:

- The IO tables are updated to the reference year – Version 10, 2014.[4]
- The IO tables are adjusted to match the trade, protection, energy, and macro-economic variables in the global datasets.
- Changes in stocks are eliminated.

This adjustment procedure of fitting the I-O tables has been named after the program FIT that implements it. FIT applies entropy-theoretic methods to adjust an I-O table to various external constraints derived from the international data sets. The fitting procedure is applied after disaggregating the primary I-O tables and constructing composite tables for each country in a composite region. Thus the inputs into the procedure are a complete set of national I-O tables,

[4] The reference year of the IO table does not need to be the same reference year in the final GTAP Data Base. Bringing all IO tables, from different base years, to a common reference year is the first adjustment performed to the country data.

with all the 65 GTAP sectors, for 244 countries, and a set of international data sets and the outputs are the fitted I-O tables and an adjusted energy volumes data set.

The following targets are applied:

i. from constraints imposed by the GTAP data base structure: zero values for changes in stocks of domestic product and imports, by commodity;

ii. from the macroeconomic data set: values at purchasers' prices for GDP, aggregate private consumption, government consumption, and investment;

iii. from the trade data set, modified according to the energy data set: border values of exports and imports, by commodity;

iv. from the protection data set, modified and supplemented from the energy data set: import duty rates, by commodity; export subsidy rates, by commodity; non-commodity indirect tax rates, by industry; commodity tax rates on intermediate usage, by industry and commodity; and rates of tax on private consumption of energy, by commodity; and

v. from the energy data set: basic values for intermediate usage of energy, by energy industry and energy commodity; basic values for private consumption of energy, by energy commodity (the energy industries and energy commodities are aggregations of standard GTAP sectors).

The FIT program incorporates an I-O quantity model, an I-O price model, and an entropy-theoretic balancing procedure. Broadly speaking, the I-O quantity model serves to remove changes in stocks and adjust exports, consumption, and investment. It feeds these final demand changes backward through the I-O structure to determine new levels for intermediate usage and primary factor employment. The I-O price model feeds tax rate changes forward through the I-O structure to adjust basic and post-tax prices for intermediate usage and final demands. The entropy procedure adjusts taste and technology variables to meet the import and energy usage targets. The general rule in the fitting procedure is to adjust the national and composite I-O tables to the international data sets, rather than the other way around, with some exceptions in agricultural domestic support and energy data.

It should be noted that since the international data sets match the data base reference year, adjusting the I-O tables to the international data sets is also the method used to update the year to the base year. This also converts the IO table into the correct units and currency.

The data assembly module is where:

i. adjustments to value added take place;

ii. the factor payments data are adjusted to incorporate land- and capital-based payments;

iii. the various international data sets and domestic data bases are put together and final checks are made;

iv. additional data such as population, capital stocks, depreciation, and savings are included; and

v. additional datasets used in the standard GTAP model or its variants (e.g., sets, elasticities, energy volumes) and summary (e.g., time series trade data, tax rates) datasets are produced.

In terms of value-added, several adjustments are made. First, labor payment data are disaggregated into skilled and unskilled labor payments using payment shares generated in the estimation procedure. Second, factor employment data for primary agriculture and natural resource-based sectors are adjusted using primary factor shares as documented in the literature. In agriculture, external estimates of factor earnings shares are used; and for natural resource-based sectors a proportion of the earnings of labor and capital is reallocated to natural resources to achieve target supply elasticities.

We further take this dataset and make modifications for the following:

1. Incorporation of IO tables of ACU member countries, which are not captured in standard GTAP Data Base, based on datasets from the UN, World Bank and IFPRI: Palestine, Lebanon, Syria, Mauritania, etc.
2. Adjustment of import tariffs to ensure that the trade agreements concluded after 2014 and before 2020 are all taken into account in the dataset.
3. Adjustment of other taxes based on various data sources in the member countries' governments.

Baseline Preparation

We use the GTAP database 10.0 (Aguiar,A. et.al., 2019) which has a reference year of 2014. Our first step is to update it to the year 2021, using macroeconomic data on GDP, consumption,

investment, government, exports and imports, available from the World Bank and updated using an entropy optimization procedure named GTAPAdjust (Horridge, M. 2011). Data on the production of tobacco crops is obtained from the FAOSTAT and that on exports and imports of tobacco crops is extracted from the Commodity Trade Statistics of the United Nations. Input-output (IO) statistics to understand the production structure of tobacco products and raw tobacco were developed based on a combination of GTAP 10 Data Base that contains aggregates IO data for the sectors "Other Crops" (ocr) and "Beverages and Tobacco Products" (b_t) and India's IO table for the year 2015-16, which is the latest available. GTAP Data Base is a comprehensive global economic dataset constructed by assembling and reconciling data components from different data sources across the world, primarily publicly available from international organizations and governments. This is documented by various authors in the literature, including a detailed description and a brief technical updated documentation. We have briefly summarized it in Appendix 1. India's IO table has information on raw tobacco and tobacco product sectors separately. Own-price elasticity of cigarettes, bidis and smokeless tobacco is taken from the published Indian literature.

Because the study is focused on India, the 141 countries and regions in the model is aggregated into two regions- India and the Rest of the World and since it is essential to focus on unmanufactured tobacco and other products in the tobacco industry, it is required that the following two sectors in GTAP - Beverages and Tobacco (b_t), Other Crops (ocr) be disaggregated. Since the data components are from different sources and need not agree with each other, an important step after collection of the data is to assemble them together and split the GTAP sector "ocr" into "raw tobacco" and "other crops", and to split the GTAP sector "b_t" into beverages, bidis, cigarettes and smokeless tobacco. For this, we follow a procedure called "MSplitCom" (Horridge, 2005), which is a GTAP commodity-splitting algorithm[42]. Broadly speaking, this procedure entails the use of entropy theoretic methods to split the GTAP sectors based on the information provided. Therefore, the model now accommodates 69 sectors of which 65 sectors are already mapped GTAPAgg sectors and four new sectors are the result of disaggregate function. However, the exact data targets are not typically met because of the inconsistencies between the external data sources and GTAP data. For example, the total production of "b_t" sector may be 1 billion USD for a country, but the actual data for tobacco products alone may be 1.1 billion USD and beverages may be around another 1.1 billion USD. In such a case, the program would assume that both beverages and tobacco products may be half of the 1 billion USD total of "b_t sector, because the external data sources suggest that

they are both equal. To rectify such problems, we carry out another procedure called GTAPAdjust. In this procedure, we re-apply all the targets mentioned in the previous paragraph for raw tobacco and all tobacco products – namely, production, IO data, exports and imports and rebalance the whole dataset so that all the supply and demand conditions are met. This is also based on entropy optimization theories.

The intuition of CGE models is based on an input-output structure where one single shock works its way through all inputs and prices to the outputs, employment, wages, prices, etc. A shock may be introduced in the form of a given percentage reduction in consumption of tobacco use. Similar analyses have been done earlier for other countries such as Tanzania[18], but they only focus on tobacco products as a group, without further disaggregation and they only consider a couple of prevalence reduction scenarios in a static sense, translating prevalence reduction into consumption reduction directly.

In this sense, the model is usually time-agnostic in a way, though we may consider the results of the scenarios as instantaneous if the policies are also assumed to be implemented instantaneously. However, for the simulation on consumption reduction as done in this report, we assume the prevalence reduction to happen in 5 years, from 2021 to 2026, and hence the results also may be interpreted to happen in this five-year period. The health benefits from the above policy in terms of the number of deaths averted and consequent benefits to the economy coming from increased labor supply are also calculated over the same time frame. As in John et.al[27], we assumed the price elasticity to be -0.91, -0.35 and -0.88 for bidi, cigarette and raw tobacco leaves respectively. The income elasticity for bidi is assumed to be 0.39, that for cigarettes is assumed to be 1.98 and for tobacco leaves, it is 0.33.

SCENARIO DESIGN

To model the consumption reduction, we assume all the consumption variables of tobacco sectors, captured by the variables *qpd (private consumption of domestic products), qpm (private consumption of imported products), qfd (Firms' intermediate input consumption of domestic products), qfm (Firms' intermediate input consumption of imported products), qgd (government consumption of domestic products) and qgm (government consumption of imported products),* to be shocked, by swapping it with the corresponding tax variables, which are denoted by the same names above, with 'q' replaced by 't'. In other words, the taxes adjust endogenously to take the shock to consumption implied by prevalence change, population change and income change. We keep the GDP variable '*qgdp*', output *'qo',* prices *'pm',*

employment *'qfe',* and all other prices and quantities in the model endogenous. For the tax scenarios, we keep all these quantities endogenous and instead hold all the tax variables exogenous and shock them.

The targeted reductions in consumption are achieved through taxation. This taxation is applied in two different ways in the model.

a. Tax on consumption
b. Tax on raw tobacco

Depending on the type of tax imposed the targeted consumption reduction has different impacts.

a) If the implicit tax is assumed to be applied to the consumption

All the variables discussed in this section are in percent change form, unless otherwise specified. Total consumption is the sum of consumption by households from domestic (*qpd*) and imported sources (*qpm*) and government (*qgd* and *qgm*). So in this scenario, our shock is effectively for *qpd* and *qpm* in the GTAP model for bidi, smokeless and cigarettes as per the estimated decline in consumption calculated above. We also shock *qgd* and *qgm*, but since the government is not a major consumer of tobacco, this is not an important shock and hence we do not discuss this here for the sake of brevity.

In the equations below, '*i*' denotes the commodity, '*s*' is the region/country. '*i*' in this model represents the Bidi, Cigarette, and Smokeless products and '*s*' represents India. *qpd* is the private consumption demand for domestic goods. It is determined by two aspects; aggregate private consumption that includes both domestic and imported commodities and the substitution between domestic and imported goods, based on the elasticity of substitution based on relative price changes in domestic with respect to overall prices. We may roughly interpret the former as the expansion effect and the latter as the substitution effect.

$$qpd\ (i,s) = qp\ (i,s) + ESUBD(i) * [pp\ (i,s) - ppd\ (i,s)] \qquad (6)$$

qp (i,s) - private household demand for commodity i in region s.
pp (i,s) - private consumption price for composite commodities (domestic + imported)
ppd (i,s) - domestic price of i consumed by private households in s.
ESUBD(i) - Substitution parameter of domestic/imported commodities for all agents.

The domestic private consumption is calculated as a function of total private consumption (*qp*) and private consumption prices - where *pp* is the aggregate price and *ppd* is the domestic consumption price. *qpm* is the private consumption demand for imported goods. Similar to qpd, this is also determined by two aspects; aggregate private consumption that includes both domestic and imported commodities and the substitution between imported and domestic goods, based on the elasticity of substitution based on relative price changes in imported commodities with respect to overall prices.

qpm(i,s) = qp(i,s) + ESUBD(i) * [pp(i,s) - ppm(i,s)] **(7)**

ppm(i,s) - price of imports of i demanded by private households in s

The private consumption of imported commodities is calculated as a function of total private consumption (*qp*) and private consumption prices - where pp is the aggregate price and ppm is the consumption price of imports. All the above-mentioned prices, pp, ppd and ppm are linked to market prices. First of all, the private consumption prices are determined as an initial consumption value-weighted aggregation of the domestic and import prices.

pp(i,s) = PMSHR(i,s) * ppm(i,s) + [1 - PMSHR(i,s)] * ppd(i,s) **(8)**

PMSHR (i,s) - share of imports for private households at agent's prices in total consumption by private households.

ppd (i,s) is the price of domestic i to private households in s.

ppd(i,s) = atpd(i,s) + pm(i,s) **(9)**

atpd is the actual tax on domestic traded commodity i purchased by private households in region r. *pm* is the market price of domestic commodity i in region r.

ppm (i,s) is the price of imports of i by private households in s and is linked to market prices as follows.

ppm(i,s) = atpm(i,s) + pim(i,s) **(10)**

pim is the market price of imported commodity i in region r.

b) If the implicit tax is assumed to be applied only on the tobacco leaf which is a major input in the production of all the three products

Because in this scenario, implicit tax is considered to be applicable only on the raw tobacco leaves, we shock the variables qfd and qfm, that are associated with the consumption of the commodity (raw tobacco leaves) by firms. *qfd (i,j,s)* considers consumption demand for a domestic commodity i by industry j in region s. Here i refers to the raw tobacco leaves; j to each of the tobacco firms that produce bidi, cigarette and smokeless products; s refers to the destination region, which here in our model is India.

qfd is the firm's consumption demand of a domestic commodity. Similar to what we discussed in the context of private consumption, this is also determined by two aspects; aggregate firm consumption that includes both domestic and imported commodities and the substitution between domestic and imported tobacco used in production, based on the elasticity of substitution based on relative price changes in domestic with respect to overall prices of tobacco used in production. We may roughly interpret the former as the expansion effect in overall consumption of tobacco in production and the latter as the substitution effect between domestic and imported tobacco.

$$qfd(i,j,s) = qf(i,j,s) - ESUBD(i) * [pfd(i,j,s) - pf(i,j,s)] \quad (11)$$

qf (i,j,s) is the aggregate demand for commodity i for use by firm j in region s
pf(i,j,s) is the firm's price for commodity i to be used in firm j in region s
pfd(i,j,s) is the firm's price for domestic commodity i to be used in firm j in region s

The consumption demand for domestic commodity by firms is calculated as a function of the total consumption demand for a commodity (i) to be used as an input in a firm (j) and consumption prices of the commodity - where pfd is the demand price for the domestic commodity (i) and pf is the aggregate demand price for the commodity i to be used in firm j.

qfm (i,j,s) considers consumption demand for imported commodity i by industry j in region s.

$$qfm(i,j,s) = qf(i,j,s) - ESUBD(i) * [pfm(i,j,s) - pf(i,j,s)] \quad (12)$$

qf (i,j,s) is the aggregate demand of commodity i for use by firm j in region s
pf(i,j,s) is the firm's price for commodity i to be used in firm j in region s
pfm(i,j,s) is the firm's price for imported commodity i to be used in firm j in region s

qfm is the firm's consumption demand of an imported commodity. Again, this is also determined by two aspects; aggregate firm consumption that includes both domestic and imported commodities and the substitution between domestic and imported goods, based on the elasticity of substitution based on relative price changes in imported commodities with respect to overall prices.

The consumption demand for imported commodity by firms is calculated as a function of the total consumption demand for a commodity (i) to be used as an input in a firm (j) and consumption prices of the commodity - where pfm is the demand price for the imported commodity (i) and pf is the aggregate demand price for the commodity to be used in firm (j).

pf(i,j,s) = FMSHR(i,j,s) * pfm(i,j,s) + [1 - FMSHR(i,j,s)] * pfd(i,j,s) **(13)**

pfd(i,j,s) - demand price for domestic commodity i by firms in sector j of region s

pfm(i,j,s) - demand price for imported commodity i by firms in sector j of region s

FMSHR - share of firms imports in domestic composite, agent's prices

All the prices, *pf*, *pfd* and *pfm* are linked to market prices as given below.

pfd(i,j,s) = tfd(i,j,s) + pm(i,s) **(14)**

tfd(i,j,s) - tax on domestic commodity i purchased by j in s

pm(i,s) - market price of commodity i in region s

pfm(i,j,s) = tfm(i,j,s) + pim(i,s) **(15)**

tfm(i,j,s) - tax on imported commodity i purchased by j in s

pim(i,s) - market price of imported commodity i in region s

A Sample statement to shock the consumption of raw tobacco leaves that are fed as input to bidi is given below.

shock qfd("rawtob","bidi","Ind") = uniform -6.88;

shock qfm("rawtob","bidi","Ind") = uniform -6.88;

The aggregate private consumption by households of each of tobacco and its products (qp) is determined by the income effect and (own and cross) price effect, based on their corresponding elasticities:

qp(i,s) = EY(i,s)*y(s) + sum(j,TRAD_COMM,EP(i,j,s)*pp(j,s)) **(16)**

EY(i,s) – income elasticity of commodity i in region s

EP(I,j,s) – Price elasticity of demand of commodity I with respect to price j in region s

y(s) –income in region s

pp(j,s) –price of private consumption of commodity j in region s.

While the model simulation directly gives the resulting changes in production, consumption, employment etc. both at the macroeconomic and sectoral level the resulting impact in terms of deaths averted were estimated outside of the model.

REFERENCES

1. Hertel, T.W., Ed. (1997) Global Trade Analysis: Modeling and Applications. Cambridge University Press, Cambridge.
2. Horridge, M. SplitCom (Victoria University, Melbourne, 2005); http://www.copsmodels.com/splitcom.html
3. Horridge, Mark. 2011. "A program to balance or adjust a GTAP database." Centre of Policy Studies, Monash University, Melbourne, Australia. https://www.copsmodels.com/archivep.htm#tpmh0104.
4. Global Trade Analysis Project. 2018. "GTAP Models: Current GTAP Model." https://www.gtap.agecon.purdue.edu/models/current.asp
5. Aguiar, Angel, Maksym Chepeliev, Erwin L. Corong, Robert McDougall, and Dominique van der Mensbrugghe. 2019. "The GTAP Data Base: Version 10." Journal of Global Economic Analysis 4(1): 1–27. https://www.jgea.org/ojs/index.php/jgea/article/view/77
